# Navigating Life Post-emergency Laparotomy: A Narrative Review on Quality-of-Life Outcomes

**DOI:** 10.7759/cureus.60583

**Published:** 2024-05-19

**Authors:** Akansha Hatewar, Chanrashekhar Mahakalkar, Shivani Kshirsagar, Sparsh Dixit, Srinivasa Reddy

**Affiliations:** 1 General Surgery, Jawaharlal Nehru Medical College, Datta Meghe Institute of Higher Education and Research, Wardha, IND

**Keywords:** multidisciplinary care, psychological distress, postoperative complications, surgical outcomes, quality of life, emergency laparotomy

## Abstract

An emergency laparotomy is a life-saving surgical procedure performed to address acute abdominal conditions. While crucial for immediate survival, this procedure can have significant long-term implications for patients' quality of life. This comprehensive review examines the physical, psychological, and social outcomes following emergency laparotomy, highlighting the importance of addressing quality-of-life concerns in this patient population. Key findings reveal that patients may experience complications, psychological distress, and challenges in social functioning post-procedure. Age, gender, and access to support networks influence outcomes. Recommendations for clinical practice include routine assessment of quality of life, multidisciplinary care, and patient education. Further research is needed to understand predictors of poor outcomes and evaluate interventions to improve quality of life post-emergency laparotomy. Healthcare providers can enhance patient care and outcomes in this vulnerable population by addressing these issues.

## Introduction and background

An emergency laparotomy is a surgical procedure performed to address acute abdominal conditions that require immediate intervention to save a patient's life or prevent serious complications. It involves making an incision into the abdomen to gain access to the internal organs for diagnostic and therapeutic purposes [1]. Common indications for emergency laparotomy include severe abdominal trauma, acute bowel obstruction, perforated viscus, and uncontrollable intra-abdominal bleeding [2].

While emergency laparotomy is often crucial for saving lives, the impact of the procedure extends beyond immediate survival. Patients who undergo emergency laparotomy may experience significant physical, psychological, and social challenges during their recovery and beyond [3]. Therefore, understanding and assessing the quality-of-life (QoL) outcomes following an emergency laparotomy is paramount. QoL outcomes encompass various aspects of an individual's well-being, including physical health, psychological well-being, social relationships, and overall satisfaction with life [4].

This review aims to provide a comprehensive examination of the QoL outcomes associated with emergency laparotomy. By synthesizing existing research and clinical evidence, this review aims to shed light on the long-term consequences of emergency laparotomy and identify factors that may influence patients' QoL post-procedure. Additionally, this review will explore the available tools and methodologies for assessing QoL outcomes in this patient population and discuss potential interventions and support services to improve patient outcomes. Ultimately, this review seeks to inform healthcare providers, researchers, and policymakers about addressing QoL concerns in patients who have undergone emergency laparotomy.

## Review

Understanding emergency laparotomy

Indications and Procedures

The indications for emergency laparotomy encompass a broad spectrum of conditions that require urgent surgical intervention. These include hernias with obstruction or gangrene, bowel ischemia, appendicitis, intestinal perforation, and obstruction [4,5]. Emergency laparotomy is frequently conducted in non-trauma, non-vascular emergency general surgery cases to explore the abdomen for various underlying pathologies [5]. The procedures involved in emergency laparotomy vary depending on the specific condition being addressed. They may entail exploratory surgery to identify and treat intra-abdominal pathologies, resection of gangrenous bowel segments, repair of perforations, and relief of obstructions [4,5]. Furthermore, emergency laparotomy in obstetric patients may involve addressing intra-abdominal bleeding post-cesarean section or postpartum hemorrhage. In contrast, in patients with malignancies, procedures may focus on managing conditions such as intestinal obstruction and perforation [4]. Emergency laparotomy encompasses a range of surgical procedures aimed at promptly addressing acute abdominal pathologies to enhance patient outcomes and reduce morbidity and mortality rates.

Risks and Complications

Emergency laparotomy poses a substantial risk, particularly among older patients, due to underlying comorbidities, frailty, and diminished physiological reserves, resulting in a notable mortality rate [6]. Complications following emergency laparotomy can significantly impact patient outcomes, encompassing abdominal infections, pulmonary issues, surgical site infections, wound dehiscence, bleeding, pneumonia, urinary tract infections, and renal complications [7-10]. Research indicates a considerable incidence of complications post-emergency laparotomy, comprising both operation-related and medical complications, which can detrimentally affect prognosis, lengthen hospital stays, and heighten short-term mortality rates [8,10]. Notably, abdominal infections, pulmonary complications, respiratory issues, and infections emerge as the primary categories of complications, with respiratory complications being the most prevalent medical complication [7,8]. Furthermore, the procedure may entail long-term consequences, prompting the necessity to scrutinize extended mortality rates and outcomes among patients undergoing emergency laparotomy [10]. A comparative analysis contrasting emergency and elective laparotomy has revealed a higher frequency of postoperative complications in emergency cases, frequently including pyrexia, nausea and vomiting, wound infections, respiratory tract infections, and other related issues [9].

Recovery Process

Implementing Enhanced Recovery After Surgery (ERAS) protocols in emergency laparotomy has demonstrated effectiveness in improving postoperative outcomes, reducing hospital stays, and lowering morbidity rates [3,11]. Research indicates that patients managed under ERAS protocols experience shorter hospital stays than those receiving conventional care, enhancing postoperative outcomes [11]. Moreover, ERAS protocols have been linked to decreased incidence of major postoperative complications such as urinary tract infections and chest infections, thereby facilitating improved recovery trajectories [11]. Providing patients with comprehensive communication regarding their diagnosis, postoperative expectations, and guidance on postoperative care is pivotal for fostering successful recovery [12]. Ensuring seamless continuity of care and timely access to healthcare professionals post-surgery is crucial for addressing any emerging concerns or complications during the recovery phase [12]. Promoting early mobilization, implementing effective pain management strategies, and tailoring rehabilitation programs are indispensable for facilitating physical recovery and reinstating functionality [13]. Equally important is offering clear dietary guidance, particularly for patients with stomas or bowel resections, and facilitating access to appropriate food choices during recovery to promote overall well-being [12]. Addressing mental health issues such as anxiety, depression, and loss of confidence post-surgery is paramount, as these factors can significantly influence a patient's recovery trajectory and overall QoL [12].

QoL assessment tools

Overview of Commonly Used Tools

Several assessment instruments are commonly utilized to evaluate QoL across diverse populations and contexts. The SF-36 (Medical Outcomes Study Short-Form 36) is one of the most frequently employed generic QoL assessment tools [14]. It comprehensively measures eight domains: physical functioning, role-physical, bodily pain, general health, vitality, social functioning, role-emotional, and mental health. Another widely utilized instrument is the EuroQol EQ-5D, which assesses five dimensions: mobility, self-care, usual activities, pain/discomfort, and anxiety/depression, supplemented by a visual analog scale (EQ-VAS) for overall health evaluation [14].

The SF-12 (12-Item Short-Form Health Survey) is a shorter version of the SF-36, focusing on measuring physical and mental health summary scores [14]. For individuals with mental illness, the Wisconsin Quality of Life Index (W-QLI) offers a tailored assessment tool encompassing physical health, psychological well-being, social relationships, functional roles, and life satisfaction [15]. The Quality-of-Life Scale (QOLS) is another versatile tool that evaluates five conceptual domains, including material and physical well-being, relationships with others, social and civic activities, personal development and fulfillment, and recreation, suitable for application across diverse patient groups and cultures [16].

Moreover, the Global QoL Scale provides respondents with the flexibility to apply their weighting system to various facets of life, offering an overall judgment of the QoL on a scale from 0 (indicating a state comparable to death) to 100 (representing the perfect QoL) [17]. These assessment tools vary in their focus, domains assessed, and intended populations, providing diverse options for evaluating QoL in various contexts. The selection of an appropriate instrument should be guided by the specific research or clinical requirements [14].

Selection Criteria for QoL Measurement

Assessing the QoL entails considering objective, subjective, and integral perceptions, which are vital for evaluating an individual's well-being and overall QoL [18]. These criteria serve as fundamental pillars in determining the holistic assessment of an individual's QoL, incorporating tangible measures and subjective experiences. The intended purpose of a QoL scale is to guide its optimal properties, distinguishing between longitudinal and cross-sectional applications. Scales tailored for longitudinal studies, such as clinical trials, possess distinct characteristics compared to those designed for cross-sectional assessments. Researchers must carefully weigh factors such as item selection, population demographics, treatment variations, response options, and the avoidance of floor and ceiling effects based on the intended use of the scale [19].

Aligning the selection of a QoL scale with its intended purpose is paramount, whether for research endeavors or clinical practice [19]. They understand the nuanced differences between scales suitable for longitudinal tracking versus those for cross-sectional comparisons, which aids in selecting the most fitting tool for the desired application, ensuring accurate and meaningful assessments. QoL instruments should exhibit discrimination capabilities between patient groups of varying severity levels. A diverse range of items within the scale enables effective differentiation between mild and severe patient populations, thereby capturing nuanced differences in QoL across diverse health conditions and patient cohorts [19]. Incorporating patient opinions is a crucial aspect of QoL measurement, emphasizing the significance of patient-reported outcomes. Patient perspectives serve as invaluable supplements or replacements for instruments developed solely by experts, fostering a more patient-centered approach to QoL assessment [20]. By prioritizing patient insights, the assessment process becomes more holistic and reflective of individual experiences, enhancing the relevance and accuracy of QoL evaluations.

Limitations and Challenges in Assessment

Patient compliance presents a significant challenge in QoL assessments, particularly with multiple and lengthy assessments administered across multiple follow-up visits [21]. The potential for decreased compliance raises concerns regarding data collection accuracy and the reliability of obtained results, highlighting a critical aspect of ensuring robust study outcomes. Variability in the selection of QoL instruments, characterized by differing scoring ranges, algorithms, and interpretations, poses a considerable obstacle to result comparison across studies [22]. This variation complicates the understanding and interpreting of QoL data, underscoring the need for standardized approaches to enhance data consistency and comparability.

The design of questionnaires holds paramount importance in fostering patient compliance and eliciting quality responses [21]. Factors such as question relevance, logical sequence, ease of comprehension, and avoidance of controversial items significantly influence patient willingness to participate and provide accurate responses, necessitating careful consideration in questionnaire development. Prolonged assessments with a high question volume may induce patient fatigue, potentially compromising response quality [21]. Beyond a certain threshold, survey fatigue can impede data accuracy and completeness, underscoring the importance of balancing assessment length with the depth of information sought.

Striking a balance between the utility of QoL data and the additional burden it imposes on patients and healthcare providers presents a notable challenge [22]. Integrating QoL assessments into clinical practice necessitates consideration of time constraints and workload, particularly in busy clinical settings, where efficiency is paramount. The psychometric properties of QoL instruments utilized in studies may vary in validation and reliability, influencing the robustness of collected data [14]. Ensuring consistent levels of validation across instruments is essential for maintaining data reliability and validity, thereby bolstering the credibility of study findings. The diverse array of instruments and questionnaire administration modes employed in studies complicates comparisons between population groups with similar characteristics or needs [14]. This impediment to comparison can compromise the validity and reliability of study outcomes, necessitating efforts to standardize assessment methodologies for enhanced data coherence and interpretability.

Physical health outcomes

Short-Term Physical Recovery

Short-term physical recovery following emergency laparotomy may exhibit variability, as certain patients may encounter a decline in their physical well-being after surgery. Factors such as complications related to stoma management, postoperative morbidity, and alterations in employment status have emerged as noteworthy contributors to the influence on physical health outcomes [23,24]. Furthermore, complications such as changes in bowel habits and issues with stoma management can exert a considerable social and psychological toll on patients, thereby impacting their short-term physical recuperation [24]. Addressing these factors and delivering comprehensive postoperative care are essential steps to optimize short-term physical recovery outcomes for individuals undergoing emergency laparotomy.

Long-Term Physical Complications

Patients undergoing emergency laparotomy often present with frailty and significant comorbidities, predisposing them to a heightened risk of postoperative complications that can adversely affect long-term outcomes [10]. Approximately one-third of these patients may undergo a decline in their physical health status after emergency laparotomy, underscoring the substantial impact on their physical well-being [7,25]. Postoperative complications, both operation-related infections and medical-related respiratory issues, are prevalent occurrences following emergency laparotomy and can precipitate severe consequences, including elevated mortality rates [8,10]. Various factors contribute to an increased susceptibility to complications, including low albumin levels, heightened surgical urgency, excessive alcohol consumption, advanced age, and elevated American Society of Anesthesiologists (ASA) class [8,10]. Furthermore, the specific diagnosis necessitating laparotomy can exert a notable influence on postoperative QoL, with procedures such as Hartmann's potentially resulting in life-altering ramifications, such as stoma management [25].

Impact on Daily Functioning and Activities

The impact of emergency laparotomy on daily functioning and activities is profound, with evidence suggesting that patients may undergo a deterioration in their psychosocial and physical health status following surgery, thereby prompting changes in employment status for some individuals [26]. The research underscores that the nature of the diagnosis necessitating laparotomy can markedly influence the postoperative QoL, with specific procedures such as Hartmann's procedure imposing life-altering consequences such as stoma management, which in turn can impede daily activities and functioning [27]. Furthermore, the presence of underlying malignancy warrants consideration, as patients with malignancies may encounter compromised physical function within the initial year post-surgery, potentially hampering their engagement in routine activities [27]. Moreover, complications arising after emergency laparotomy can significantly compromise patients' prognoses and extend hospital stays, thereby affecting their capacity to resume normal daily activities [8]. These complications, whether operation-related or medical, may precipitate a decline in physical health status, consequently restricting patients' ability to carry out daily functions and activities [8].

Psychological impact

Post-Traumatic Stress Disorder (PTSD)

The psychological ramifications of emergency laparotomy on patients, particularly concerning PTSD, present a significant concern. While research dedicated explicitly to PTSD following emergency laparotomy is limited, studies suggest that patients may grapple with psychological distress and trauma after such a life-threatening event [23,28]. Findings from one study revealed that nearly one-third of patients experienced some form of psychological impact during the early postoperative period, with 5.3% reporting feelings of nervousness and 31.6% reporting anxiety [28]. Additionally, another study underscored that patients undergoing emergency laparotomy often encounter a decline in their psychosocial and physical health status, a phenomenon that can exacerbate psychological distress [23].

Various factors can influence the psychological impact of emergency laparotomy, including the severity of the surgical procedure, the patient's preoperative mental health status, and the quality of postoperative care. However, further research is warranted to grasp emergency laparotomy's psychological effects comprehensively and devise effective interventions to alleviate these effects [23,28]. Regarding PTSD specifically, data on its prevalence among patients following emergency laparotomy remain limited. Nevertheless, given the traumatic nature of the event and the potential for enduring psychological repercussions, it is conceivable that some patients may develop PTSD. Consequently, additional research is imperative to deepen our understanding of emergency laparotomy's psychological impact and formulate targeted interventions to support patients throughout their recovery journey [23,28].

Anxiety and Depression

The impact of emergency abdominal surgery on the patient's mental health, particularly concerning anxiety and depression, has garnered considerable research attention. Studies indicate that undergoing emergency surgery can precipitate psychological distress, with a notable proportion of patients experiencing negative emotions during the initial postoperative period [28]. Factors such as the nature of the surgery (major vs. non-major) and the duration of postoperative hospitalization have been linked to elevated levels of anxiety and depression among patients recovering from emergency abdominal surgery [28,29]. Depression, recognized as a significant factor influencing outcomes across various medical conditions, assumes particular importance in the context of abdominal surgery. However, the precise extent of its impact in this specific setting warrants further investigation [30]. Additionally, research has delved into the association between mental illness and outcomes following emergency surgery, encompassing a range of general surgical procedures. This underscores the critical need to comprehend the intricate interplay between mental health and surgical outcomes [31]. By elucidating these relationships, clinicians and researchers can develop more effective strategies to support patients' mental well-being throughout the surgical process and enhance overall treatment outcomes.

Coping Mechanisms and Support Systems

The psychological repercussions of emergency laparotomy on patients, encompassing coping mechanisms and support structures, constitute a crucial facet of postoperative care. The research underscores that nearly one-third of patients encounter psychological impacts in the early postoperative phase, with factors such as stoma complications, postoperative morbidity, and alterations in employment status significantly influencing health outcomes [28]. Patients undergoing major surgery, particularly those with prolonged recovery periods, often contend with more pronounced psychological challenges, underscoring the imperative for formal psychological support for individuals undergoing emergency procedures [28]. Comprehending the psychological hurdles confronted by patients post-emergency laparotomy is indispensable for delivering comprehensive care. Coping strategies may vary among individuals but could encompass seeking professional psychological assistance, participating in support groups, fostering open communication with healthcare providers, and enlisting the support of family and friends for emotional reinforcement. Establishing a robust support network comprising healthcare professionals, family members, and friends is pivotal in assisting patients in navigating the psychological ramifications of emergency surgery and facilitating their recovery journey.

Social and emotional well-being

Relationships and Social Interactions

The experience of emergency laparotomy significantly influences patients' relationships and social interactions. Research indicates that individuals who experience social isolation, strained relationships, and neglect of personal needs are more susceptible to adverse post-surgery outcomes [32]. Emphasizing the importance of social relationships and support in the recovery process, particularly among older adults who rely on these connections for effective participation in multimodality care pathways, is crucial [33]. Studies suggest enhanced social integration, including increased contacts and interactions within a patient's social network, correlates with improved postoperative function and QoL [33]. Furthermore, offering practical social support has proven effective in facilitating home-based health behavior changes among frail older adults, enhancing social functioning and general health [33]. The impact of emergency laparotomy on patients' social and emotional well-being is multifaceted, influencing not only their immediate recovery but also their long-term QoL. Patients undergoing emergency laparotomy often encounter challenges transitioning into a chronic disease state, resulting in life-altering changes that affect their social connections, mental health, and overall well-being [4]. Understanding and addressing these social and emotional aspects are integral to providing comprehensive care and supporting patients throughout their recovery.

Work and Financial Implications

The ramifications of emergency laparotomy on patients extend beyond health outcomes to encompass work and financial considerations. Research underscores that emergency laparotomy can significantly affect patients' employment status and financial well-being post-surgery. A study examining the patient-reported impact of emergency laparotomy on employment and health status one year after surgery revealed that factors such as stoma complications, postoperative morbidity, and alterations in employment significantly influenced health status [23]. Moreover, investigations into the financial risks associated with emergency abdominal surgery, including emergency laparotomy, have shed light on the economic burden patients face. Research focused on the financial ramifications of emergency abdominal surgery underscores the substantial direct medical expenditures associated with specific procedures, such as emergency laparotomy, underscoring the financial strain experienced by patients undergoing these surgeries [34]. These findings highlight the importance of considering the broader socioeconomic impact of emergency laparotomy and underscore the need for comprehensive support measures to address the financial implications for patients.

Adjusting to Life Changes

Adjusting to life changes can be a formidable process that affects individuals emotionally, socially, and mentally. Whether these changes are positive or negative, they can disrupt familiar routines and trigger stress, anxiety, and even depression. Recognizing the signs of adjustment disorder, a transient condition stemming from significant life changes, is crucial, prompting individuals to seek appropriate support and coping strategies to navigate these transitions adeptly [35-37].

To adapt to life changes healthily, individuals can employ various strategies. Prioritizing self-care, maintaining social connections, and managing change at their own pace are essential components of this process. Seeking professional help, if necessary, can provide valuable guidance and support [35-37]. Engaging in activities that foster well-being, such as staying physically active and incorporating relaxation techniques like mindfulness or deep breathing, can aid in the adjustment process.

Furthermore, understanding the impact of change on mental health and well-being is paramount. Stress resulting from life changes may manifest in physical and mental symptoms such as headaches, insomnia, depression, and anxiety. Acknowledging these effects and implementing healthy coping mechanisms, such as positive reframing and acceptance of the situation, can empower individuals to navigate transitions effectively while safeguarding their mental health [38].

Factors influencing QoL

Age and Gender

The interplay between age and gender significantly influences various facets of individuals' lives, impacting their QoL and encounters with discrimination. The research underscores that age and gender can affect the subjective QoL differently. While statistical analyses may not consistently reveal disparities in QoL between men and women, age has been consistently linked to satisfaction levels, with older individuals often reporting higher life satisfaction levels than their younger counterparts [39]. Furthermore, the experiences of older women with disabilities illuminate the compounded challenges they confront due to discrimination, bias, and marginalization. These individuals must be more noticed and represented in development policies, programs, legislation, and humanitarian efforts. This underscores the intersectionality of gender, age, and disability in shaping individuals' experiences and opportunities [40]. Understanding the intricate dynamics of how age and gender intersect is pivotal for addressing inequalities, fostering inclusivity, and enhancing the well-being of individuals across diverse age groups and gender identities. By acknowledging and confronting the unique challenges faced by individuals based on their age and gender, stakeholders can collaborate toward creating more equitable and supportive environments that cater to the diverse needs and experiences of all individuals.

Pre-existing Health Conditions

Pre-existing health conditions influence patients' outcomes and QoL undergoing emergency laparotomy. The research underscores that individuals subjected to emergency laparotomy frequently present with significant comorbidities, intensifying the risk of postoperative complications and unfavorable outcomes [8,10,27]. Older age and the presence of comorbidities are intricately linked to an augmented burden of postoperative complications, prolonged hospital stays, and an elevated mortality risk after emergency laparotomy [8,27]. Studies elucidate that pre-existing health conditions, such as high ASA classification and elevated Charlson Comorbidity Index (CCI) scores, emerge as pivotal factors associated with poor long-term outcomes and heightened mortality following emergency laparotomy [10,27]. These conditions can impede the recovery trajectory, heighten the likelihood of postoperative medical complications, and contribute to an escalated risk of mortality during the follow-up period [27]. Recognizing and addressing these pre-existing health conditions are paramount for optimizing patient outcomes and enhancing the overall efficacy of emergency laparotomy interventions.

Support Networks and Access to Healthcare

**Figure 1:**
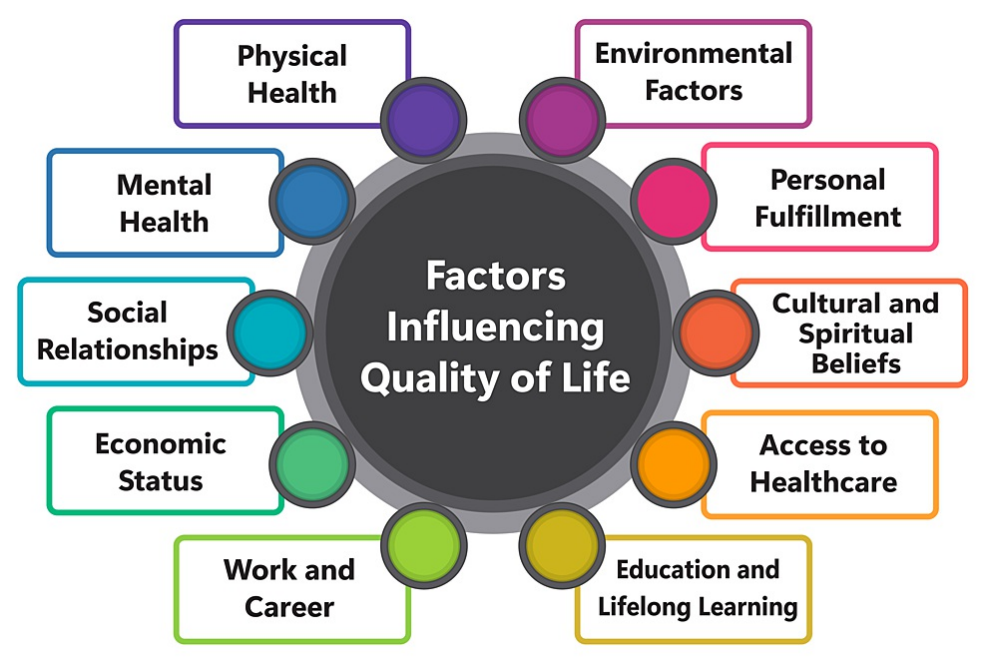
Factors influencing quality of life The image was created by the corresponding author.

The role of support networks in facilitating access to healthcare emerges as a pivotal focus in the provided sources. The research underscores social support as a versatile resource that aids individuals in navigating the intricacies of the healthcare system and fulfilling institutional expectations for effective engagement in healthcare processes [41]. This support assumes particular significance for families grappling with the care of seriously ill children, empowering them to balance diverse responsibilities, oversee medical care, and cultivate constructive relationships with healthcare providers [41]. Furthermore, the sources elucidate the broader ramifications of social support on healthcare access, extending beyond mere health insurance status. They highlight the necessity for policies and interventions aimed at assisting parents in managing the myriad demands associated with caring for a seriously ill child, encompassing financial obligations, medical care coordination, and safeguarding family well-being [41]. Initiatives aimed at augmenting access to high-quality healthcare should consider the varying capacities of parents in managing their children's medical needs and the pivotal role of social support in alleviating the burdens families encounter within healthcare environments [41]. By recognizing and bolstering the supportive networks available to families, healthcare systems can effectively enhance access to care and alleviate the challenges inherent in managing the care of seriously ill children. Figure 1 shows the factors influencing QoL.

Future directions and research opportunities

Improving QoL​​​​​​​ Measurement Tools

The role of support networks in facilitating access to healthcare emerges as a pivotal focus in the provided sources. The research underscores social support as a versatile resource that aids individuals in navigating the intricacies of the healthcare system and fulfilling institutional expectations for effective engagement in healthcare processes [41]. This support assumes particular significance for families grappling with the care of seriously ill children, empowering them to balance diverse responsibilities, oversee medical care, and cultivate constructive relationships with healthcare providers [41]. Furthermore, the sources elucidate the broader ramifications of social support on healthcare access, extending beyond mere health insurance status. They highlight the necessity for policies and interventions aimed at assisting parents in managing the myriad demands associated with caring for a seriously ill child, encompassing financial obligations, medical care coordination, and safeguarding family well-being [41]. Initiatives aimed at augmenting access to high-quality healthcare should consider the varying capacities of parents in managing their children's medical needs and the pivotal role of social support in alleviating the burdens families encounter within healthcare environments [41]. By recognizing and bolstering the supportive networks available to families, healthcare systems can effectively enhance access to care and alleviate the challenges inherent in managing the care of seriously ill children [42,43].

Identifying Risk Factors and Predictors

Several factors contribute to increased mortality post-emergency laparotomy, including age, ASA status, preoperative sepsis, dependency status, current cancer, and comorbidities [44]. Additionally, the recognized risk factors for postoperative mortality encompass preoperative anemia, heightened bleeding risk, hypotension, azotemia, coagulopathy, and surgical delay [44,45]. Perioperative variables play a crucial role in influencing postoperative morbidity and mortality among emergency laparotomy patients. Factors such as preoperative hemodynamic parameters, ASA status, and unplanned escalation to postoperative intensive care are associated with increased morbidity and mortality rates [46]. Furthermore, intraoperative parameters such as ASA status and unplanned escalation to intensive care units independently correlate with heightened postoperative morbidity [46]. The UK National Emergency Laparotomy Audit (NELA) has developed a risk-predictive tool for mortality following emergency laparotomy, demonstrating good calibration and accuracy in predicting 30-day mortality rates [47]. Combining this tool with the modified frailty index (mFI) and nutritional status can further refine its predictive accuracy [47].

Developing Targeted Interventions

Enhancing preoperative risk assessment and care is a pivotal strategy in influencing outcomes, potentially impacting patient results significantly. This involves refining patient selection criteria, optimizing preoperative management strategies, and customizing interventions to suit individual patient needs [3,48,49]. Implementing multimodal perioperative care pathways and integrating evidence-based practices such as goal-directed therapy and intensive postoperative care is another critical approach to improving outcomes [3,48]. Minimizing delays to surgery and ensuring prompt diagnosis and intervention represent critical steps in reducing morbidity and mortality rates [3,48]. Ensuring the involvement of specialist surgeons and anesthetists, coupled with fostering interdisciplinary collaboration, holds promise in enhancing patient outcomes by providing more holistic care [3,48]. Tailoring care pathways to specific patient cohorts, such as those with intestinal obstruction or perforation, is essential for addressing their distinct challenges and requirements [3]. Strategies to optimize nutritional support and promote early mobilization are instrumental in fostering recovery and mitigating complications [3]. Improving organizational aspects of management, such as the implementation of standardized care pathways and enhanced recovery protocols, can further contribute to favorable outcomes [48].

## Conclusions

In conclusion, this comprehensive review highlights the multifaceted impact of emergency laparotomy on patients' QoL. The findings underscore the importance of considering the immediate surgical outcomes and the long-term physical, psychological, and social consequences for patients undergoing this procedure. Healthcare providers must integrate routine assessments of QoL outcomes into postoperative care protocols to identify at-risk patients and provide tailored support. Collaborative efforts involving multidisciplinary teams are crucial in addressing the diverse needs of patients post-emergency laparotomy. Furthermore, ongoing research is needed to longitudinally evaluate QoL outcomes, develop standardized assessment tools, and assess the effectiveness of interventions to improve patient well-being. By addressing these research gaps and implementing evidence-based practices, clinicians can enhance patients' overall care and outcomes in navigating life post-emergency laparotomy.
